# Correlation Between Thromboelastography and Standard Coagulation Tests at a Tertiary Community Hospital

**DOI:** 10.7759/cureus.94347

**Published:** 2025-10-11

**Authors:** Daniela Fernandez, Melissa Santibañez, Angel Maldonado, Bradley Rogers

**Affiliations:** 1 Medical Science – Diabetes, Novo Nordisk, Plainsboro, USA; 2 Pharmacy Practice, Nova Southeastern University, Barry and Judy Silverman College of Pharmacy, Fort Lauderdale, USA; 3 Critical Care Pharmacy, Memorial Regional Hospital, Hollywood, USA; 4 Clinical Pharmacy, Memorial Regional Hospital, Hollywood, USA

**Keywords:** coagulation tests, hemostasis and thrombosis, hospital and clinical pharmacy, point of care testing, thromboelastography (teg)

## Abstract

Introduction: Thromboelastography (TEG) assesses the clotting capacity of whole blood in real time, while standard coagulation tests (SCTs) represent static measures. Evidence of correlation between values obtained via TEG and SCTs remains scarce. The purpose of this study was to evaluate the correlation between TEG and SCT values and assess interventions received utilizing an institution-specific TEG algorithm.

Materials and methods: This was a single-center retrospective chart review. Data were obtained using the TEG Manager® program and the EPIC® electronic medical record for adult patients ≥18 years admitted between July 2019 and September 2019. TEG values assessed included R-time, K-time, maximum amplitude (MA), and lysis at 30 minutes. SCT values assessed included PT/INR (prothrombin time/international normalized ratio), platelet count, fibrinogen, and hemoglobin/hematocrit. Descriptive statistics were used for the primary analysis. Spearman's coefficient was used for correlation analysis, with strong correlation defined as r>0.7.

Results: The primary analysis included 100 patients, with a median age of 62.5 years (IQR 49.3-76.0) and 52% male. No strong positive correlations were identified between any TEG and SCT values. Twenty-two patients (22%) received a blood product, and six patients (6%) received a hemostatic agent, with the majority receiving platelets or four-factor prothrombin complex concentrate. An inappropriate intervention was identified in 30% of TEGs, of which 22 (61.1%) indicated a need for platelets due to a low MA angle without subsequent platelet administration.

Conclusions: The lack of strong positive correlations between TEG and SCT values was consistent with the existing literature. Further provider and pharmacist education is required to improve utilization of the institutional TEG algorithm.

## Introduction

Thromboelastography (TEG) is a viscoelastic method of assessing the clotting profile of whole blood in real time [1]. This hemostatic assay can analyze clot formation, kinetics, strength, stability, and degradation [1,2]. In this way, TEG technology can produce real-time images of patient-specific clot formation and breakdown. TEG parameters typically include R-time, kinetic (k)-time, alpha angle, maximum amplitude (MA), and lysis at 30 minutes (Ly30) [2,3].

TEG is increasingly being utilized as a point-of-care assessment of a patient’s clotting status. It has been successfully implemented as a point-of-care test and used to guide blood transfusion strategies across several patient populations, including cardiac surgery, trauma, perioperative bleeding, obstetrics, chronic liver disease, and liver transplantation [4-7]. A Cochrane review of TEG and rotational thromboelastometry (ROTEM) reported that using viscoelastic tests to guide transfusion strategies resulted in significantly decreased blood product transfusions in bleeding patients [8]. Additionally, a randomized trial comparing a TEG-guided transfusion algorithm versus routine transfusion therapy in cardiac surgery patients after cardiopulmonary bypass found a significant decrease in postoperative transfusions in TEG patients, attributed to earlier identification of hemostatic abnormalities during the intraoperative period [9].

In comparison, standard coagulation tests (SCTs), such as activated partial thromboplastin time (aPTT), prothrombin time (PT), international normalized ratio (INR), fibrinogen, and platelet count, are static measures and thus measure isolated parts of the hemostatic process [2]. These tests are unable to evaluate the strength of clot formation or assess platelet function [10]. With the development of the TEG6s system, which automates and expedites sample processing times via the use of cartridges that can be run simultaneously, point-of-care coagulation testing within the hospital setting is expected to become more widespread [11].

Despite this evidence, there remains a scarcity of data on the correlation between reported TEG values and the SCTs used in everyday hospital practice. Correlation between TEG and SCTs may help improve the administration of appropriate treatments, including transfusions and reversal/hemostatic agents, and may possibly decrease the need for additional extraneous tests. This may, in turn, allow for more efficient and individualized evaluation of a patient's clinical status, aiding clinical decision-making, especially in acute scenarios such as the reversal of anticoagulant-associated major bleeds.

Memorial Regional Hospital (MRH) is a 700-bed tertiary community teaching hospital and Level I trauma center serving South Broward County, Florida, and is the flagship hospital of the Memorial Healthcare System. At MRH, all clinicians can order a standard TEG with physician approval. Although TEG was initially introduced in 2015 for use by cardiac perfusionists, its use was expanded to the trauma service in 2017 and subsequently to additional critical care areas, including the surgical, medical, and neurologic intensive care units. The TEG algorithm for major hemorrhage was implemented in August 2017, with the venous thromboembolism (VTE) prophylaxis algorithm subsequently implemented in March 2019, and then the novel coronavirus (COVID-19) VTE prophylaxis algorithm was implemented in 2020. TEG has also been incorporated into the MRH massive transfusion and emergent anticoagulant reversal protocols. Finally, the TEG6s system was launched in late 2024.

The purpose of this study was to evaluate the correlation between TEG values and SCTs at a community teaching hospital following implementation of a TEG algorithm and to assess the interventions received by patients as guided by this algorithm.

## Materials and methods

Study design

**Figure 1:**
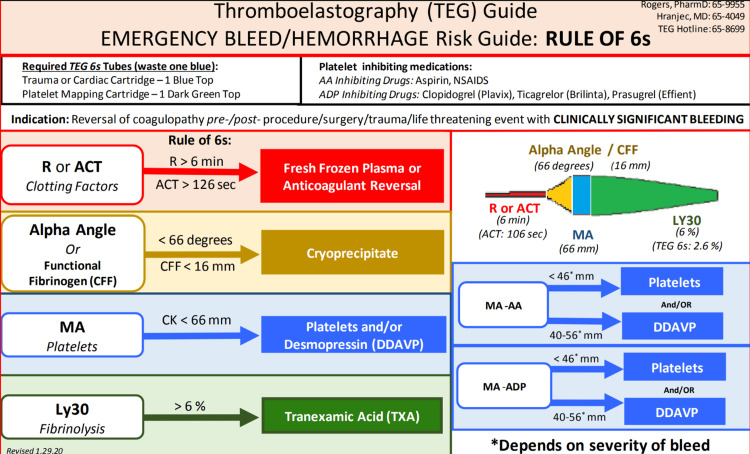
Institutional TEG algorithm The image is reproduced with permission from the Memorial Regional Hospital Venous Thromboembolism (VTE) Prophylaxis Algorithm. Abbreviations: TEG = thromboelastography.

This was a single-center, retrospective chart review study. Data were obtained using the hospital's TEG Manager® program and EPIC® electronic medical record. Figure 1 shows the institutional TEG algorithm used at MRH.

Patient selection

Included patients were adults 18 years of age or older with a TEG drawn at the hospital between July 2019 and September 2019 and with SCTs drawn within four hours before or after their TEG. This four-hour window was selected based on the institution's turnaround time for the tests to be ordered, drawn, and processed by the lab at similar time points, taking into consideration how a change in the patient's clinical status could impact the results of the tests if drawn at drastically different time points.

Patients were excluded if either the SCTs or TEGs were drawn after an intervention (defined as administration of a blood product and/or reversal/hemostatic agent). Blood products included packed red blood cells (PRBCs), platelets, fresh frozen plasma (FFP), and cryoprecipitate. Reversal/hemostatic agents included four-factor prothrombin complex concentrate (4F-PCC, Kcentra), idarucizumab, vitamin K, desmopressin (DDAVP), and tranexamic acid.

Endpoints

The primary endpoint of this study was the correlation between TEG and SCT values, conducted as an assessment for any correlation between all available SCT and TEG values. TEG values assessed included R-time, K-time, MA, and lysis at 30 minutes. SCT values assessed included PT/INR, platelet count, fibrinogen, and hemoglobin/hematocrit. The secondary endpoint was interventions administered in response to TEG results based on the MRH institution-specific TEG algorithm.

Statistical analysis

Descriptive statistics were used in the primary analysis. Categorical variables were reported using frequencies and percentages, while continuous variables were reported using medians and interquartile ranges. A correlation analysis was conducted using Spearman's correlation test. A strong correlation was defined as an r > 0.7, and an alpha of 0.05 was statistically significant. All analyses were completed using IBM SPSS Statistics for Windows, Version 26 (Released 2019; IBM Corp., Armonk, New York).

Use of artificial intelligence (AI)

No components of this study were developed or reviewed by generative AI.

## Results

Baseline characteristics

**Figure 2:**
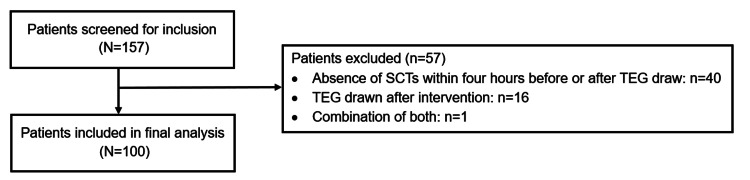
Patient screening Abbreviations: SCT = standard coagulation tests; TEG = thromboelastography.

A total of 157 patients were screened for inclusion. Fifty-seven patients were excluded for the absence of SCTs four hours before or after TEG draw (n=40), TEG drawn after an intervention (n=16), or a combination of both (n=1). Ultimately, 100 patients were included in the primary analysis. Figure 2 shows the patient flow process.

Most included patients were male (n=52, 52%), with a median age of 62.5 years (IQR 49.3-76.0). The most frequent diagnoses were organ transplant evaluation (n=26, 26%), trauma (n=23, 23%), and brain hemorrhage (n=18, 18%). Additional diagnoses, as well as pre-hospitalization use of anticoagulants and antiplatelets, are summarized in Table 1.

**Table 1:** Baseline characteristics *Other diagnoses included hematuria (n=2), pre-/post-partum complications (n=2), ventral hernia (n=1), altered mental status (n=1), sepsis (n=1), pulmonary embolism (n=1), abdominal wall hematoma (n=1), acute liver failure (n=1), nephrolithiasis (n=1), rectal bleeding (n=1), symptomatic anemia (n=1), syncope (n=1), and gastrointestinal bleeding (n=1). Data are represented as frequencies with percentages or medians with interquartile ranges. Abbreviations: DAPT = dual antiplatelet therapy.

Variable	Value
Age, in years	62.5 (49.5–76.0)
Male sex	52 (52)
Diagnosis	
Transplant evaluation	26 (26)
Trauma	23 (23)
Brain hemorrhage	18 (18)
Other*	15 (15)
Cardiac	10 (10)
Transplant surgery and complications	8 (8)
Pre-hospitalization anticoagulant use	n=22
Apixaban	9 (41.0)
Warfarin	6 (27.3)
Rivaroxaban	5 (22.7)
Dabigatran	2 (9.1)
Pre-hospitalization antiplatelet use	n=39
Aspirin	27 (69.2)
Clopidogrel	6 (15.4)
DAPT	6 (15.4)

Primary endpoint: correlation between TEG and SCTs

Correlation analysis between TEG values and SCTs is described in Table 2. Overall, R-time values positively correlated with PT and INR (p=0.010 and p=0.007, respectively). K values also positively correlated with PT/INR. Alpha angle and MA value were both positively correlated with platelet count (p<0.0001). No correlation was found between Ly30 and any of the SCTs evaluated. Of note, fibrinogen correlated only with MA, while platelet count correlated with MA, alpha angle, and K; however, none of these associations represented a strong correlation.

**Table 2:** Correlation analysis between standard coagulation test (SCT) and thromboelastography (TEG) values Abbreviations: aPTT=activated partial thromboplastin time; Hb=hemoglobin; Hct=hematocrit; INR=international normalized ratio; K=clot formation; Ly30=percent decrease in clot amplitude at 30 min; MA=maximum amplitude; PT=prothrombin time; R=reaction time; TEG=thromboelastography.

Variable		Thromboelastography (TEG) values				
		R	k	Alpha angle	MA	Ly30
		min	min	(α)	mm	%
Standard coagulation test (SCT) values	Platelet count	-0.171 (p=0.108)	-0.455 (p<0.0001)	0.438 (p<0.0001)	0.568 (p<0.0001)	0.058 (p=0.576)
	aPTT	-0.496 (p<0.0001)	-0.276 (p=0.021)	-0.319 (p=0.007)	-0.190 (p=0.115)	-0.042 (p=0.731)
	PT	0.312 (p=0.010)	0.446 (p<0.0001)	-0.449 (p<0.0001)	-0.461 (p<0.0001)	-0.012 (p=0.074)
	INR	0.332 (p=0.007)	0.398 (p=0.0006)	-0.407 (p=0.0004)	-0.459 (p<0.0001)	-0.149 (p=0.256)
	Fibrinogen	0.083 (p=0.843)	0.059 (p=0.888)	0.100 (p=0.810)	0.639 (p=0.071)	0.091 (p=0.833)
	HCT	0.013 (p=0.899)	0.165 (p=0.111)	-0.157 (p=0.288)	-0.087 (p=0.403)	0.022 (p=0.831)
	Hb	0.041 (p=0.701)	0.187 (p=0.069)	-0.184 (p=0.074)	-0.099 (p=0.340)	0.032 (p=0.762)

Secondary endpoint: appropriateness of therapeutic interventions

A total of 49 interventions were administered based on TEG results. Among these, 22 patients received at least one blood product; a total of 33 transfusions were received, including platelets (n=19), PRBCs (n=8), FFP (n=3), and cryoprecipitate (n=3). A total of six reversal agents were administered: 4F-PCC (n=4), idarucizumab (n=1), and intravenous vitamin K (n=1). Two patients received both a blood product and a reversal agent.

Among all interventions assessed against the institutional TEG algorithm, 30% were determined to be inappropriate. A detailed summary of reasons for inappropriate interventions is presented in Table 3. Notably, over 70% of patients had a low MA angle, indicating that either platelets should be transfused or DDAVP should be administered, per the institutional TEG algorithm. However, a platelet transfusion was neither ordered nor completed, and these patients also did not receive DDAVP.

**Table 3:** Inappropriate interventions Data are represented as frequencies with percentages.

Reasons for Inappropriate Intervention	Frequency (n=36)
Reversal agent not indicated	3 (8.3)
Blood product not indicated	3 (8.3)
Platelets	1 (33.3)
Plasma	1 (33.3)
Cryoprecipitate	1 (33.3)
Blood product indicated but not administered	30 (83.3)
Platelets	22 (73.3)
Plasma	5 (16.7)
Cryoprecipitate	3 (10.0)

## Discussion

Available TEG assays and common TEG parameters are summarized in Table 4.

**Table 4:** Summary of thromboelastography (TEG) assays, values, and management strategies Adapted from [1-3].

TEG Assays	Description
TEG citrated kaolin (CK)	Intrinsic pathway activated assay
TEG with heparinase	Eliminates the effect of heparin in the blood sample
Rapid TEG (rTEG)	Intrinsic and extrinsic pathways are activated; allows for a more rapid assessment of coagulation
TEG Platelet mapping (PM)	Includes receptors involved in platelet function (e.g., adenosine diphosphate, arachidonic acid); identifies the level of platelet inhibition and aggregation in a blood sample
TEG Values	Description
R time	Time to first detected clot formation; represents standard clotting factors; if prolonged, consider plasma, prothrombin complex concentrates, or reversal of causative anticoagulant
Kinetic (k) time	Clot formation and strength; if prolonged, consider cryoprecipitate or fibrinogen concentrate
Alpha angle	Clot propagation speed; if reduced, consider cryoprecipitate or fibrinogen concentrate
Maximum amplitude (MA)	Maximum clot strength; correlates to platelet function; if reduced, consider platelets and/or desmopressin
Lysis at 30 minutes (Ly30)	Corresponds to fibrinolysis; if prolonged, consider tranexamic acid or aminocaproic acid

Among the available published literature on TEG correlation with SCTs, the patient populations and study methods have generally been heterogeneous and limited in their external validity. Comparison of TEG with SCTs in 180 surgical patients with localized prostate cancer identified a strong positive correlation (r=0.844) between R and PT/INR, a strong positive correlation (r=0.8302) between K-time and PT/INR, and weak positive correlations between alpha angle and MA with fibrinogen levels [12]. A prospective correlation analysis of 60 Swedish surgical patients at high bleeding risk found moderate agreement between TEG and SCTs for measures of hypocoagulability, with an overall sensitivity of 33% (95% CI 19%-52%) and specificity of 95% (95% CI 87%-98%) [6]. Sensitivity to detect thrombocytopenia (platelet count <150 x10-9 cells/L) with TEG was 17% (95% CI 7%-36%) using alpha angle and 25% (95% CI 11%-45%) using MA; sensitivity to detect fibrinogen deficiency (fibrinogen <2 g/L) was 11% (95% CI 3%-29%) using alpha angle and 21% using MA (95% CI 8%-43%) [6].

Another study in non-bleeding ICU patients identified a borderline strong correlation (r=0.680) between MA and platelet count, a positive correlation between MA and fibrinogen, and a positive correlation between R and K values with aPTT [13]. An analysis of 40 elderly Chinese patients with long-bone fractures revealed a hypercoagulable state compared to control patients, with lower K values serving as significant predictors of elevated fibrinogen levels [14]. This study also identified positive correlations between fibrinogen and MA/alpha angle, platelet count and MA, and aPTT and R value [14]. Beyond the adult critically ill population, a weak correlation between TEG heparinase and aPTT, as well as between MA and platelet count, has been identified in pediatric patients receiving extracorporeal membrane oxygenation [15].

While the existing literature has mostly shown weak positive correlations between specific SCTs and specific TEG values across heterogeneous populations, our intent was to run correlation analyses between all SCT and all TEG values to determine relationships within our patient population. Our findings of positive correlations between R and K values with PT/INR, as well as MA with platelet count, were expected per the existing literature. Most of the included patients in our study were surgical, trauma, or cardiac, all of which are populations for which these correlations have been supported in the published literature. The lack of correlation between MA and fibrinogen in our study may have been attributed to inconsistent ordering of fibrinogen levels, as only nine patients (9%) had a fibrinogen level ordered; therefore, it is difficult to determine a reliable correlation on this parameter.

Various studies have also shown that TEG can reduce the number of blood transfusions in patients with bleeding abnormalities [5,8]. However, the ITACTIC trial recently reported no differences in overall outcomes between trauma patients who were resuscitated with a massive hemorrhage protocol when guided by viscoelastic tests versus SCTs [16]. Considering the larger proportion of trauma patients in our study (23%), future evaluation should involve an assessment of TEG's impact on transfusion strategies and patient outcomes within our institution, in addition to a cost analysis to assess if utilizing TEG is a cost-effective modality.

Furthermore, in acutely ill patients with severe chronic liver disease, one study in 34 adults with Child-Pugh C chronic liver disease found that both TEG and SCTs identified hypocoagulability via delayed clot formation and decreased thrombus strength [17]. Furthermore, a Korean study of 123 cirrhotic, 52 non-cirrhotic, and 84 healthy adults revealed hypocoagulability among the cirrhotic group and a weak correlation between R value and PT/INR (r=0.173) [7]. Clinically, patients with normal TEG parameters did not require transfusions [7]. These data may suggest that critically ill chronic liver disease patients are not actually in the state of hemostasis that is conventionally asserted [7,17]. While the nature of TEG allows for the distinction between normal versus hypercoagulable or hypocoagulable states, as well as the ability to provide detailed information on platelet inhibition, SCTs are unable to comprehensively provide this information. Over a quarter of TEGs analyzed in our study were drawn from patients being evaluated primarily for liver transplants; this must be acknowledged as a potential source of future research to further identify best practices in this patient population within our institution.

Although the institutional TEG algorithm demonstrated in Figure 1 also includes guidance on the use of TEG in hypercoagulability, this component is not frequently applied by hospital providers. Another area of improvement is the selection of appropriate interventions, which is why our institution-specific TEG algorithm was subsequently streamlined to provide clearer guidance on treatment options for both hypercoagulable and hypocoagulable patients. A pre-/post-study comparing interventions in these groups may help further elucidate common provider practices and areas for education as MRH continues to expand the use of TEG within the hospital.

Limitations

Several limitations were identified in this study. This was a single-center study based on retrospective chart reviews, which potentially introduced bias based on omissions or errors in reporting and documentation. The small sample size may also limit the external validity of our findings. Additionally, adjustments for multiple comparisons and potential confounders were not conducted during our analysis; as we analyzed several correlations between SCTs and TEG values within this study, this posed a risk of results potentially seeming statistically significant by chance. The three-month time period was intentionally selected as a feasibility measure, as this study was intended as an initial assessment of the clinical and safety impact of our hospital’s TEG-guided algorithm following its initial implementation.

Furthermore, our sample featured predominantly trauma and surgical/cardiac patients, which may also limit external validity to different demographics. As mentioned previously, other factors that affect coagulation (e.g., anticoagulant use, liver disease, active bleeding) have been reported in the existing literature; however, our statistical analysis plan did not factor in the impact of such factors, and future studies on institutional TEG algorithms could purposely build this into their primary analyses. Additionally, we acknowledge the limitations of allowing a four-hour window to obtain SCTs before or after the TEG. Despite this criterion, all eligible patients within our study period were included in this evaluation to minimize selection bias as much as possible. In certain patient populations, such as the critically ill, coagulation status can fluctuate rapidly. This was allowed because TEG was, at the time, a new process at our institution and thus was not common practice when assessing a patient’s hemostasis. Future evaluations of the TEG algorithm will ideally ensure samples are drawn at the same time point in the patient care process.

We also did not assess clinical outcomes or the impact of TEG on the overall number of transfusions needed. While transfusions were quantified, no comparison was made to the required number of transfusions or to the appropriateness of transfusions in patients without TEGs. Furthermore, it was identified that not all TEGs included in the primary analysis were drawn while patients were admitted; several TEGs were drawn for outpatient transplant evaluations to assess hypocoagulability and hypercoagulability prior to surgery. As such, internal validity was limited given the variation in common practices between inpatient and outpatient settings. In the assessment of interventions, the majority were appropriate per the institutional TEG algorithm. No similar comparison was made to assess appropriateness using SCTs alone due to inter-provider variability and lack of protocolized transfusion policies based on SCTs. Of the interventions that were inappropriate, the majority involved a blood product indicated per the TEG finding that was ultimately not administered to the patient. These types of deviations may be expected to an extent if accounting for providers' clinical judgment, which this study did not quantify.

## Conclusions

While no strong positive correlations were identified between TEG and SCT values, a positive correlation between R and K values with INR and between MA value with platelet count was observed. The results also indicate that the MRH institution-specific TEG algorithm was generally effectively utilized by providers, primarily among the trauma and transplant services. There remains a need for ongoing education among clinicians to both disseminate these results and explain the applications and utility of the TEG algorithm, given the recent implementation of this process at MRH.
